# The use of data science to analyse physiology of oxygen delivery in the extracorporeal circulation

**DOI:** 10.1186/s12872-019-01301-6

**Published:** 2019-12-13

**Authors:** Marceli Lukaszewski, Rafal Lukaszewski, Kinga Kosiorowska, Marek Jasinski

**Affiliations:** 1 Department of Anaesthesiology and Intensive Therapy, Wroclaw Medical University, Borowska 213, 50-556 Wroclaw, Poland; 2 Accenture Sp z o.o, Warsaw, Poland; 3 Department and Clinic of Cardiac Surgery, Wroclaw Medical University, Wroclaw, Poland

**Keywords:** Extracorporeal circulation, Goal-directed perfusion, Data science, Oxygen delivery, Pump flow, Haemodynamics

## Abstract

**Background:**

Recent scientific reports have brought into light a new concept of goal-directed perfusion (GDP) that aims to recreate physiological conditions in which the risk of end-organ malperfusion is minimalized. The aim of our study was to analyse patients’ interim physiology while on cardiopulmonary bypass based on the haemodynamic and tissue oxygen delivery measurements. We also aimed to create a universal formula that may help in further implementation of the GDP concept.

**Methods:**

We retrospectively analysed patients operated on at the Wroclaw University Hospital between June 2017 and December 2018. Since our observations provided an extensive amount of data, including the patients’ demographics, surgery details and the perfusion-related data, the Data Science methodology was applied.

**Results:**

A total of 272 (mean age 62.5 ± 12.4, 74% male) cardiac surgery patients were included in the study. To study the relationship between haemodynamic and tissue oxygen parameters, the data for three different values of DO_2_i (280 ml/min/m^2^, 330 ml/min/m^2^ and 380 ml/min/m^2^), were evaluated. Each set of those lines showed a descending function of CI in Hb concentration for the set DO_2_i.

**Conclusions:**

Modern calculation tools make it possible to create a common data platform from a very large database. Using that methodology we created models of haemodynamic compounds describing tissue oxygen delivery. The obtained unique patterns may both allow the adaptation of the flow in relation to the patient’s unique morphology that changes in time and contribute to wider and safer implementation of perfusion strategy which has been tailored to every patient’s individual needs.

## Background

The emergence of extracorporeal circulation (ECC) has been a milestone in the history of heart surgery. Throughout the years, the ECC procedure of commencing the heart-lung machine and switching off the heart together with a pulmonary circulation from the bloodstream has been the gold standard of the majority of cardiac surgery procedures. Nonetheless, the ECC procedure is associated with significant postoperative complications, occurring either de novo or as an exacerbation of existing organ dysfunctions. It may also influence the coagulation system and trigger the vasoplegic syndrome [1]. Recently, much attention has been devoted to the detrimental effects of inadequate organ perfusion on cardiopulmonary bypass (CPB) [2]. The current widely accepted CPB management strategies are based on simplified schemes including regulation of core perfusion parameters, thus not restoring the patient’s unique physiological circulation. The pump flow rate usually remains constant and is set on 2.2–2.4 l/min/m^2^ [3]. The perfusion pressure that optimizes transmembrane transport is maintained above 50 mmHg, which is the lowest value of cerebral blood flow autoregulation [4]. The problem of ECC management is aggravated by the lack of EBM-based standards covering basic haemodynamic parameters, i.e., pump flow rate and perfusion pressure, as well as haemodilution range or indications for the vasoconstrictors use. This has led clinicians to adapt different ranges of defined core parameters for guiding the perfusion throughout cardiac surgical units worldwide [1, 2, 4].

A haemodynamic patient – heart-lung machine model aims to study the disparity between the patient’s unique haemodynamics during the ECC procedure and CPB settings. Currently applicable perfusion protocols are based solely on physiological haemodynamic parameters, such as CI or perfusion pressure. Consequently, they apply the same average parameters to each patient, which does not seem adequate since the initiation of the heart-lung machine changes the physiological haemodynamic conditions. Therefore, the implementation of non-invasive real-time monitoring of oxygen transport and haemodynamics has become an intrinsic factor for the newly introduced concept, the so-called Goal-Directed Perfusion (GDP). The aim of this concept is to restore the physiological conditions of cell and tissue respiration during the ECC by optimizing both tissue oxygen delivery and extraction. The evidence proving the effectiveness of this concept is very limited, with only a handful of articles demonstrating favourable clinical impact on the reduction of CPB-related complications, such as kidney or CNS injury. The oxygen supply threshold (DO_2_), below which the deterioration of end-organ function can be observed, was set above 272 ml/m^2^/min [5, 6]. Additionally, new parameters determining adequate oxygen transport, such as percentage oxygen saturation of venous blood (SvO_2_), oxygen consumption (VO_2_), ratio of oxygen consumed to oxygen delivered (O_2_ER) and oxygen delivery indexed to body surface area to carbon dioxide production indexed to body surface area ratio (DO_2_i/VCO_2_i) were adopted. Yet the DO_2_i/VCO_2_i ratio often remains blurred due to frequent CO_2_ insufflations into the operating field for the anti-embolic purposes.

$$ {\mathrm{DO}}_2=\mathrm{pump}\ \mathrm{flow}\ \mathrm{x}\ \left[\left(\mathrm{Hg}\ \mathrm{x}\ \mathrm{Sat}\ \mathrm{x}\ 1,36\right)+\left(0.003\ \mathrm{x}\ \mathrm{PaO}2\right)\right] $$

DO_2_ is directly proportional to the oxygen content in the blood and, therefore, the pump flow rate enables the adjustment of these two parameters to obtain satisfactory DO_2_ value. Perfusion pressure depends on the cardiac output and the vascular tone, whereas on CPB it depends mainly on the flow rate and blood viscosity. Systemic vascular resistance, as usually practiced, can be modified through vasoactive agents [7].

The aim of our observational study was to find physiological relationships between the patient’s haemodynamics and the heart-lung machine settings that would then allow to introduce the perfusion tailored to the individual patient’s needs and to spread the concept around the world.

## Methods

We performed a retrospective analysis of 272 consecutive cardiac surgery patients operated on at the Wroclaw University Hospital between June 2017 and December 2018. Each procedure was conducted with the use of the S5 Heart-Lung Machine (*LivaNova PLC, London, UK*) equipped with the System M real-time non-invasive monitoring *(Spectrum Medical, Gloucester, UK*). All the procedures were carried out according to the ward standards in normothermia. The patients’ agreement for treatment and data collection was approved by the local Bioethics Committee.

### Data collection and statistical analysis

The dataset of 272 cases, which amounts to over 351 thousand rows of data, was collected from both electronic medical records and the VIPER data management system, where all the perfusion-related data were collected. Demographic data were expressed as mean values with either a standard deviation (SD) for continuous variable data or as a percentage of the total for categorical data (Table 1). We compared: patient baseline characteristics with operative details obtained from electronic medical records, results of sensors in-line monitoring and pump flow rate measurements. An enormous amount of data and the diversity of the data pattern determined the use the Data Science calculation tools [8]. According to Data Science nomenclature, our estimates are described as: structured data (e.g., laboratory results), semi-structured data (e.g., sensor data) and unstructured data (e.g., patient notes) [9–11]. Additionally, using the Data Science capabilities we looked for adequate pump flow rates for which relevant GDP conditions, such as DO_2_ > 280 ml/m^2^/min, SvO_2_ > 68% and MAP> 60 mmHg, were met. Our toolset was based in on Anaconda Distribution (Anaconda Inc., https://www.anaconda.com), Python 3.6 (Python Software Foundation, https://www.python.org) and Jupyter Notebook (Jupyter Project, https://jupyter.org). Data cleaning and analysis was performed using Pandas (Python Data Analysis Library), whereas visualization was done with Matplotlib (Matplotlib Development Team, https://matplotlib.org) and Seaborn libraries (https://seaborn.pydata.org). All those applications use Berkeley Software Distribution (BSD) type of license, which means they are free for distribution, modification, and private and commercial use and do not require any liabilities in return. All the data, both structured and unstructured, were firstly gathered in a Microsoft Excel (Microsoft office package, https://products.office.com/pl-pl/excel) format with each patient bookmarked with a separate spreadsheet. From there it was imported into a Jupyter Notebook, where, using Python Pandas module, it was merged into one big dataset that used the time for its main index. It was then scrapped from duplications and empty rows, cleaned from unnecessary information and missing information was interpolated from nearby points. Those operations were necessary to create a data platform that was subsequently investigated by adding to it different set of constrains. Finally, visual analysis was made with the use of 2D and 3D plots.

**Table 1:** Demographic characteristics of the study group

Number of patients (n)	272
Age (years), mean ± SD	62.5 ± 12.4
Gender male, n (%)	203 (73.6)
Body surface area (m^2^), mean ± SD	1.95 ± 0.21
Body mass index, mean ± SD	28.38 ± 4.78
Risk factors, n (%)	
Coronary artery disease	190 (68.8)
Hypertension	188 (68.1)
Diabetes mellitus	79 (28.6)
Hypercholesterolemia	59 (21.4)
COPD	12 (4.3)
ESRD	3 (1.1)
Type of surgery, n (%)	
CABG	133 (48,9)
Other single procedure	95 (34,9)
Double procedure	43 (15,8)
Triple procedure	1 (0.4)

*CABG* Coronary bypass grafting, *COPD* Chronic obstructive pulmonary disease, *ESRD* End stage renal disease

## Results

**Fig. 1:**
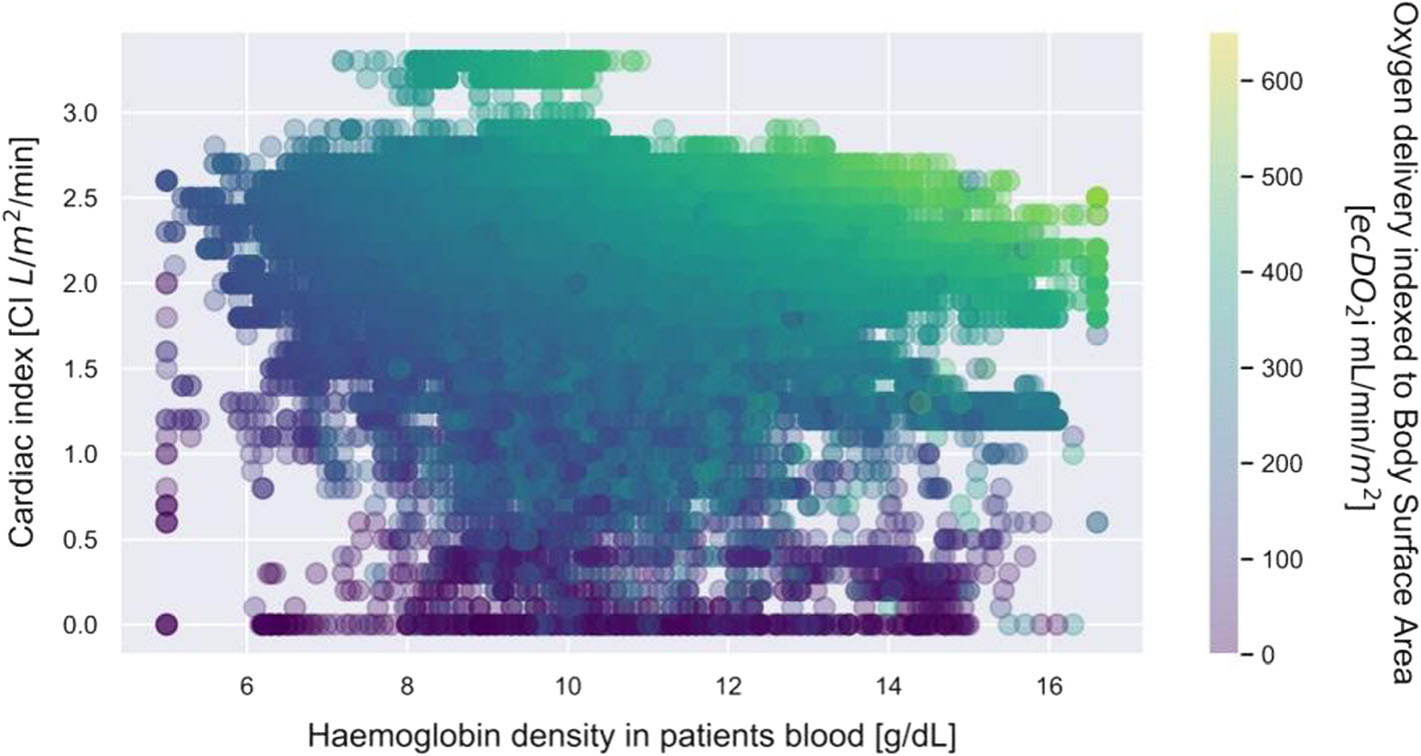
A full set of the cardiac index data during extracorporeal circulation (ECC), in the function of haemoglobin density and tissue oxygen delivery for patients with mixed venous oxygen saturation (SvO_2_) above 68% and mean arterial pressure (MAP) above 60 mmHg. Oxygen delivery (DO_2_i) has been illustrated with colour, where the darker shades correspond with lower values

**Fig. 2:**
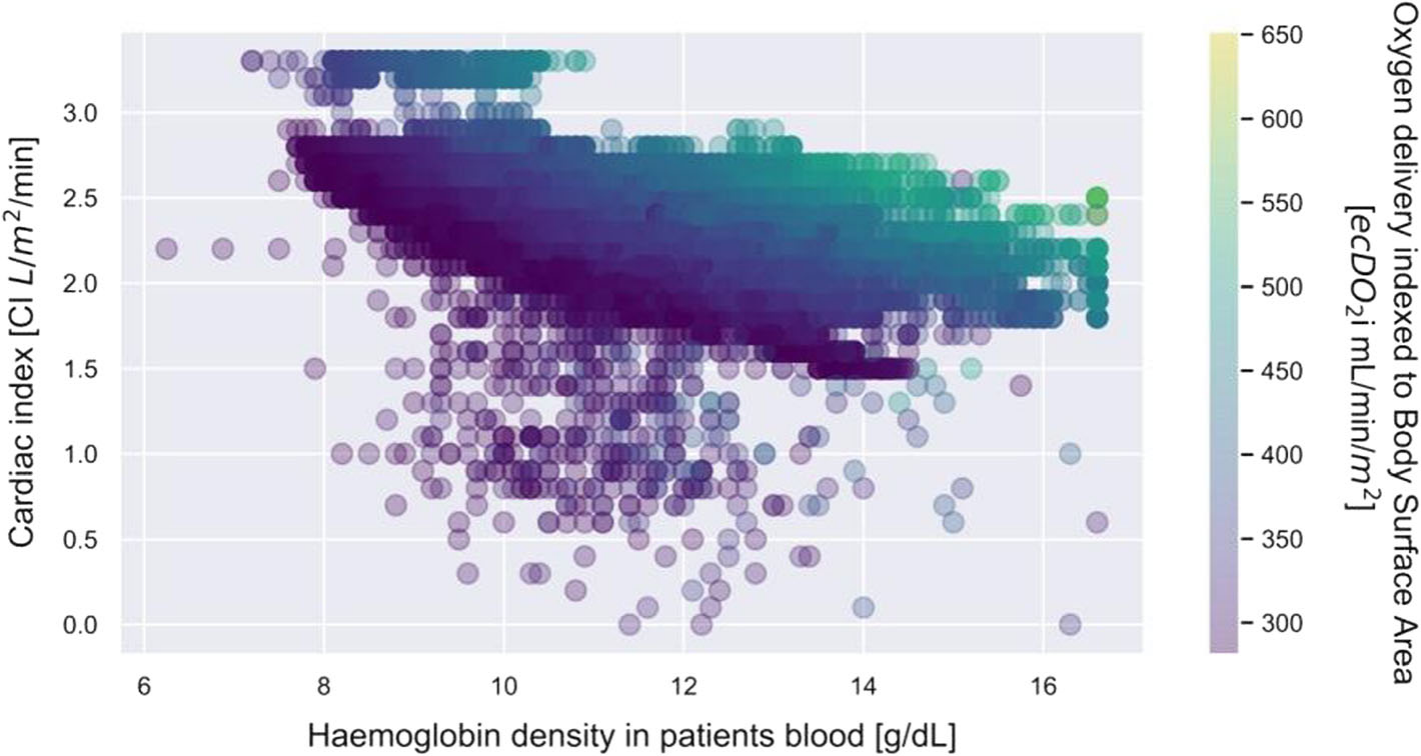
A set of the cardiac index data during extracorporeal circulation (ECC) for patients with mixed venous oxygen saturation (SvO_2_) above 68%, mean arterial pressure (MAP) above 60 mmHg and tissue oxygen delivery (DO_2_) greater than 280 mL/min/m^2^, in the function of haemoglobin density and tissue oxygen delivery

**Fig. 3:**
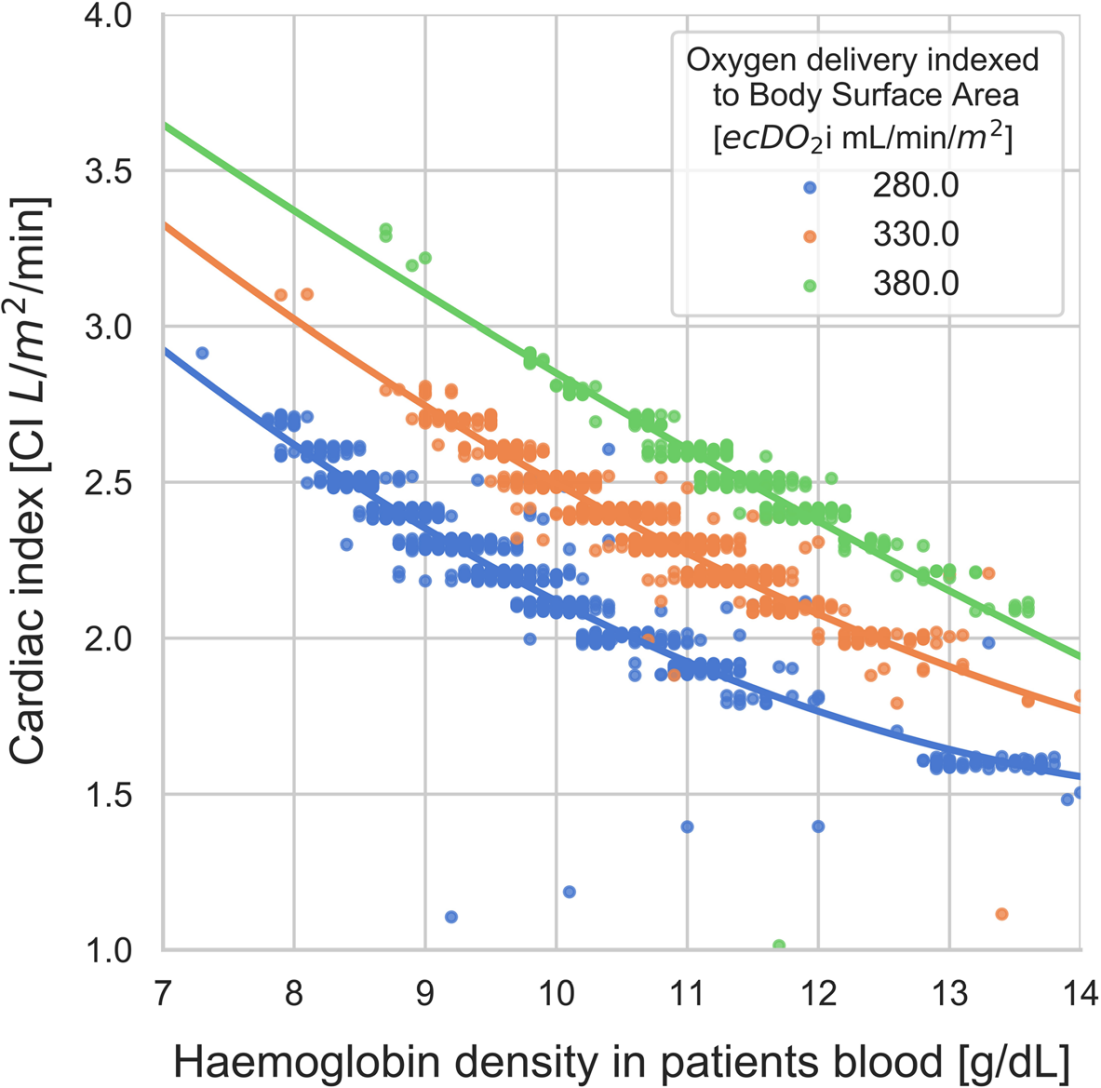
The linear regression curves for three selected oxygen delivery (DO_2_) levels showing the relationship between cardiac index (CI) and haemoglobin density

A total of 272 (mean age 62.5 ± 12.4, 74% male) cardiac surgery patients were included in this study. Nearly half of them (49.5%) underwent isolated CABG procedure, whereas 34.4% underwent another single procedure. A double procedure was conducted on 15.6% of patients. One patient underwent a triple procedure. Demographic findings of the study population are presented in Table 1. The formation of heterogeneous information derived from patient baseline characteristics along with operative details and the results of in-line real-time CPB monitoring and pump flow rate measurements created a common platform for coherent multi-modal data processing. As the methodology of Data Science enables the analysis of various types of data, the rejection of erroneous readings and the use of regression methods, we employed it to identify and analyse diagnostic and therapeutic features for the ECC. It was ensured that the database was cleared from any duplications and empty rows. The first visual analysis of the gathered data shows haemoglobin concentration and cardiac index (CI) value on x and y-axis respectively (Fig. 1). Oxygen delivery (DO_2_i) was illustrated in colour, where darker shades correspond with lower values. The image reveals a predictable pattern in whichthe DO_2_i level goes up with both Hb and CI. It also reveals that most of the measurements were taken for CI set between 1,3–2,8 L/min/m^2^ and Hb values between 9 and 12 g/dL. The database contained a moderate amount of DO_2_i values lower than 280 mL/min/ m^2^. In the majority of cases they resulted from short-term blood flow reductions from the CPB related to cardiac surgical procedures. Continuing data analysis, we carefully selected data for which the minimum value of DO_2_i was at least 280 mL/min/m^2^ and SvO_2_ was above 68%. Additionally, the impact of outlier points that go out of the pattern at random due to various measurement uncertainties was reduced by shortening the database by those rows where CI values had less than 1 hundred corresponding Hb measurements. Those new constraints left a strong visible pattern in the data (Fig. 2). The arch-shaped cut on the lower part of the scattered data in Fig. 2 corresponds to the relationship between CI and Hb for our limit DO_2_ value. To study the relation, we extracted the data for three different values (280 ml/min/m^2^, 330 ml/min/m^2^and380ml/min/m^2^) of oxygen delivery (DO_2_i), which were subsequently described with weighted linear regression of second order (Fig. 3). Each set of those lines shows a descending function of CI in Hb concentration for the set DO_2_i.

## Discussion

Recently accumulating evidence has suggested that the trends in current perfusion practice need to be improved as we experience tremendous technological improvement every day. The discussed new GDP concept provides an ideal opportunity to apply treatment which is tailored to the individual patient’s needs and, therefore, aims to diminish the risk of complications throughout the entire surgical procedure. Consequently, the emerging concept jeopardizes the current widely accepted CPB strategies of organ perfusion based on a constant pump flow rate and the ubiquitous indicators of tissue perfusion, such as lactate concentration and urine output, with lactates representing effective tissue perfusion and metabolism balance, and urine output being a vital indicator of renal perfusion. The main goal of GDP is to optimize tissue oxygen delivery and extraction. Interestingly, adequate perfusion represented by the flow rate is a crucial component of DO_2_.

In current clinical practice, the haemodynamic values used by perfusionists are based solely on observational studies designed on the basis of the physiology of a healthy individual. For instance, the pump flow rate is the equivalent of the average calculated cardiac index (CI). However, one should raise a question whether the haemodynamic parameters observed in a healthy individual can be adapted during the ECC when the heart and pulmonary circulation are switched off from the bloodstream and the tangible pump flow rate reflects the unique physiology of cardiac output only to a small extent. The advancement in System M in-line non-invasive monitoring provides an opportunity to conduct real-time observation of flow rates, both venous and arterial saturation, haemoglobin level, the extent of carbon dioxide production and also calculate all exponents of oxygen transport. The aim of our study was to find the relationships between the set pump flow rates and the ultimate GDP conditions, such as DO_2_ > 280 mL/min/m2, SvO_2_ > 68% and the minimum MAP of 60 mmHg. Statistical analysis was performed with Data Science calculation tool, which is more frequently introduced as codes that can assess with high probability if, for instance, a skin mole will turn into cancer or classify genes responsible for leukaemia [12, 13]. In our study, we strived to present how the application of this technology can help to easily visualise the correlation between factors that would be incalculable elsewhere. Using the described tools, the pattern in the data that corresponds to the relationship between CI and Hb for the same DO_2_i value was derived (Fig. 3). Hence, to ensure a certain level of DO_2_ at MAP> 60 mmHg, haemoglobin value necessitates the adjustment of the appropriate pump flow rate. The graph illustrates that at Hg of 10 g%, the initial pump flow rate within the study population was set on 2.2 l/min/m^2^, while at Hg level of 8 g% the pump flow rate should be sustained on approximately 2.6 l/min/m^2^. The described relationship is part of the GDP concept, however, the perfusion pressure above 60 mmHg is an additional parameter that was analysed.

## Conclusions

The presented observations reflect substantial mechanisms in the newly created haemodynamic patient – heart-lung machine model. Nevertheless, the depicted concept requires further observation. Appropriate models dedicated to higher perfusion pressures and different DO_2_ values should be evaluated as it would be extremely useful in managing patients concurrently affected by primary cerebral and visceral hypoperfusion in particular. The revealed relationship outlines basic values for the initial settings of the heart-lung machine to optimize perfusion during the ECC. Moreover, perfusion strategy should be individualized to match the patient’s unique physiology that changes over time.

## Data Availability

The datasets used and/or analysed during the current study are available from the corresponding author on reasonable request.

## Abbreviations

CABG: Coronary artery bypass grafting
CI: Cardiac index
CPB: Cardiopulmonary bypass
DO_2_: Oxygen delivery
DO_2_i: Oxygen delivery indexed to Body Surface Area
EBM: Evidence-based medicine
ec: extracorporeal
ECC: Extracorporeal circulation
GDP: Goal-directed perfusion
Hb: Haemoglobin
MAP: Mean arterial pressure
O_2_ER: Ratio of oxygen consumed to oxygen delivered
SVO_2_: Mixed venous oxygen saturation
VCO_2_: Carbon dioxide production
VO_2_: Oxygen consumption
